# Pharmacogenetics of TNF inhibitor response in rheumatoid arthritis utilizing the two-component disease activity score

**DOI:** 10.2217/pgs-2020-0043

**Published:** 2020-10-30

**Authors:** Syed SA Gilani, Nisha Nair, Darren Plant, Kimme Hyrich, Ann W Morgan, Andrew P Morris, Anthony G Wilson, John D Isaacs, Anne Barton, James Bluett, BRAGGSS Consortium

**Affiliations:** 1 School of Medicine, University of Manchester, Manchester, M13 9PT, UK; 2 NIHR Manchester Biomedical Research Center, Central Manchester NHS Foundation Trust, Manchester Academic Health Science Center, Manchester, M13 9WL, UK; 3 Versus Arthritis Centre for Genetics & Genomics, Centre for Musculoskeletal Research, The University of Manchester, Manchester, M13 9PL, UK; 4 Versus Arthritis Center for Epidemiology, Center for Musculoskeletal Research, The University of Manchester, Manchester, M13 9PL, UK; 5 School of Medicine, University of Leeds & NIHR Leeds Biomedical Research Centre, Leeds Teaching Hospitals NHS Trust, Leeds, LS2 9JT, UK; 6 School of Medicine & Medical Science, Conway Institute, University College Dublin, Dublin, Dublin 4, Ireland; 7 Institute of Translational & Clinical Research, Newcastle University & Newcastle upon Tyne Hospitals NHS Foundation Trust, Newcastle upon Tyne, NE1 7RU, UK

**Keywords:** biomarker, rheumatoid arthritis, single nucleotide polymorphism, TNF inhibitors, treatment response

## Abstract

**Aim:**

TNF inhibitor drugs are a treatment option for rheumatoid arthritis, but response is not universal. Response is typically measured using the composite 4-component (4C) disease activity score 28 (DAS28) which contains more subjective measures. This study used a validated 2-component (2C) DAS28 score to determine whether SNPs associated with response were replicated in the UK population.

**Materials & methods:**

A literature review identified TNF inhibitor response SNPs. Linear regression was conducted to replicate associations with 4C or 2C-DAS28 response.

**Results:**

Eighteen independent SNPs were analyzed in 1828 patients. One and four associations with 4C and 2C-DAS28 response respectively were identified (p ≤ 0.05).

**Conclusion:**

Further genetic associations were replicated using the 2C-DAS28 which may reflect the objective nature of 2C-AS28.

Rheumatoid arthritis (RA) is a heterogeneous disease ranging from mild joint inflammation (synovitis) to progressive joint damage with multisystem involvement. Distinct immunophenotypes are recognized with anticitrullinated protein antibody positive and negative disease. Treatment is based on controlling inflammation; TNF inhibitor (TNFi) medications have significantly improved patient outcomes, but response is not universal. Approximately 40% of patients will experience primary or secondary TNFi failure [1]. Time on ineffective medication may lead to irreversible joint damage and increasing healthcare costs [2]. The discovery of genetic markers of response would be a considerable advance in RA treatment, leading to more targeted therapeutic prescribing.

The heritability of RA susceptibility is estimated to be up to 65% [3], in the past decade, significant advances have been made in understanding and identifying genetic susceptibility markers with approximately 100 loci identified [4]. These studies show how genetic association can inform insight into the pathogenesis of RA, but do not have immediate clinical impact. The identification of genetic markers associating with treatment response, pharmacogenetics, potentially could be translated more quickly and several groups have conducted genome-wide and association studies to identify predictors of TNFi response (reviewed by Bek *et al.* [5]); however, replication of findings across studies has been very limited. RA treatment response can be measured in a number of ways. The National Institute for Health and Care Excellence recommends measuring TNFi response using the European Union League Against Rheumatism (EULAR) response criteria which utilize the four component (4C) disease activity score (DAS) 28 [6,7]. The 4C-DAS28 is a composite score of a 28 swollen and tender joint count (SJC and TJC), a visual analogue score (VAS: 0–100mm) of patient global assessment of arthritis activity and an objective biochemical marker of inflammation erythrocyte sedimentation rate (ESR) or C-reactive protein (CRP). The 4C-DAS28 was developed to reflect treatment decisions in the era before modern imaging techniques were undertaken in routine clinical practice and biologic therapies were not available. In the finalized 4C-DAS28 the TJC was given a greater weight than the SJC. The VAS and TJC can be elevated, however, due to illnesses other than active RA such as depression and fibromyalgia, which can lead to elevation of the 4C-DAS28 that is not due to active synovitis [8,9]. One TNFi pharmacogenetic study has evaluated the heritability of the separate components of the 4C-DAS28 and shown that the heritability of change in ESR and SJC are 48 and 39%, respectively, but when evaluating the more subjective measures the heritability of the change in TJC and VAS is nonexistent [10]. Taken together, these observations suggest that using the change in 4C-DAS28 as an outcome measure may impede the identification of genetic markers of TNFi response. More recently, a revised DAS28 score was developed in cohorts of patients with early RA with different weights applied to most accurately reflect ultrasound-determined synovitis. When considering ultrasound-confirmed synovitis, only SJC and CRP were significantly associated. This revised 2C-DAS28 was validated in additional cohorts and was demonstrated to show greater association with the progression of radiographic erosions, compared with the 4C-DAS28 [11].

The aim of the current study was to investigate the association of previously identified TNFi response SNPs to determine whether a greater number of SNPs are associated with 2C-DAS28 response compared with the original 4C-DAS28 in a large UK cohort.

## Materials & methods

### Identifying SNPs associated with TNFi response

A literature review was conducted in Medline during April 2018 to identify SNPs with previous evidence of association with TNFi response discovered using a Genome-Wide Association Study (GWAS) approach that reached a p-value of <10^-5^ or <10^-3^ and with validation in an independent cohort (p < 0.05), see Supplementary data for search terms.

### Patients

Patients commencing a TNFi were recruited prospectively to the Biologics in Rheumatoid Arthritis Genetics and Genomics Study Syndicate (BRAGGSS; www.braggss.co.uk) observational cohort study as described previously [10]. Blood samples were obtained for DNA extraction from TNFi treated patients with RA who met the following inclusion criteria: a physician diagnosis of RA; aged >18 years; self-reported Caucasian ancestry; baseline components of the 4C-DAS28 available and; starting the TNFis infliximab, etanercept, adalimumab, certolizumab or golimumab. Participants’ written consent was obtained. Ethical approval for BRAGGSS was given by the North West Ethics Research Committee (COREC 04/Q1403/37).

### Clinical & demographic data collection

Clinical measurements included SJC28, TJC28, ESR/CRP and VAS at baseline and again at follow-up (6 months). 4C-DAS28 and 2C-DAS28 were calculated as previously described [7,11]. CCP levels were measured using a commercial ELISA (Axis-Shield Diagnostics Limited, Dundee, UK). Anti-CCP levels >5 U/ml were regarded as positive as per the assay guidance. Clinico-demographic details were recorded including age, gender and current medication. Data were collected at baseline (prior to TNFi initiation) and at 6 months after TNFi initiation.

### Statistical analysis

Multivariate linear regression was performed to assess the association between each SNP genotype and absolute change in 4C-DAS28 and 2C-DAS28 following TNFi treatment at 6 months of follow-up. Regression analyses were adjusted for baseline 4C-DAS28 or 2C-DAS28 as appropriate. All SNPs associated were analyzed with reference to the minor allele. These analyses were performed using Plink statistical software (version 1.07; http://pngu.mgh.harvard.edu/purcell/plink/). Using binomial testing in R, independent SNPs (r^2^ <0.2) were investigated to evaluate whether the number of associations (p ≤ 0.05) were statistically significant or would be expected by chance alone [12]. Where SNPs were in linkage disequilibrium, the SNP with the lowest p-value identified in the literature review was taken forward for binomial testing. In addition, a sensitivity analysis of anti-CCP positive only patients was undertaken.

## Results

Thirty-six SNPs were identified in the literature review and selected for analysis, following quality control of the genotype data, 26 remained available for analysis and 18 were independent (r^2^ ≤0.2) (Supplementary Table 1). In total, 1812 patients were eligible for analysis, of which 1620 and 1643 had complete 4C-DAS28 and 2C-DAS28 6 month follow-up data respectively. Baseline clinico-demographic features are shown in Table 1. Following 6 months of TNFi treatment the mean absolute change in 2C-DAS28 and 4C-DAS28 was 2.13 and 2.46, respectively, (Supplementary Figure 1).

**Table 1.** Baseline clinico-demographic characteristics.

Baseline characteristic	n (%)	Missing (n)
Anti-CCP positive	840 (79.4)	754
Current DMARD	1483 (81.8)	0
Concurrent MTX	1185 (90.7)	506
Female	1387 (76.6)	2
Age, median (IQR)	57.7 (49.9–64.7)	0
Baseline 4CDAS28; mean (SD)	6.15 (0.95)	0
Baseline 2CDAS28; mean (SD)	4.58 (1.22)	0
TNFi		
– Infliximab	471 (26.0)	
– Etanercept	640 (35.3)	
– Adalimumab	583 (32.1)	
– Certolizumab	92 (5.1)	
– Golimumab	26 (1.4)	

CDAS: Component disease activity score; DMARD: Disease modifying antirheumatic drug; IQR: Interquartile range; MTX: Methotrexate; SD: Standard deviation; TNFi: TNF inhibitor.

In the initial multivariate analysis, using the absolute change in 4C-DAS28 over 6 months as the primary outcome measure adjusting for baseline 4C-DAS28, one SNP was associated with response (rs12081765; intergenic p = 0.01) which was also associated with 2C-DAS28 response (p = 0.04). Using 2C-DAS28 as the outcome measure, adjusting for baseline 2C-DAS28 five SNPs were associated with response (Table 2 & Supplementary Table 2). Using binomial testing, there were more significant associations with change in 2C-DAS28 at p ≤ 0.05 than would be expected by chance (p = 0.01). In comparison, the number of SNP associations with change in 4C-DAS28 at p ≤ 0.05 was not significant (p = 0.61). Restricting the analysis to anti-CCP positive patients only (n = 840) revealed one SNP was associated with 4C-DAS28 and six with 2C-DAS28 (Supplementary Table 3).

**Table 2.** Association of prior identified SNPs with the response to treatment with anti-TNF agents in the BRAGGSS cohort.

SNP	Gene	4C-DAS28 (n = 1620)		2C-DAS28 (n = 1643)	
		β	p-value	β	p-value^‡^
rs885814	*ALPL*	0.02	0.76	0.11	0.04
rs1350948^†^	*Intergenic*	-0.09	0.16	-0.13	0.04
rs12081765^†^	*Intergenic*	-0.12	0.01^‡^	-0.10	0.04
rs13393173^†^	*CERS6*	-0.01	0.91	-0.11	0.05
rs17301249^†^	*EYA4*	0.12	0.07	0.12	0.05

^†^ Included in binomial test due to linkage disequilibrium with other SNPs (r^2^ >0.2).

^‡^ P ≤ 0.05.

## Discussion

Existing therapies for RA aim to induce disease remission and control joint inflammation. No single therapy is universally effective, and a number of patients will experience a trial and error process before an effective treatment is initiated. Furthermore, pain can persist despite achieving DAS28 remission, which can be mistaken as active inflammatory disease, leading to inappropriate escalation of therapy and misinterpretation of clinical trials [13]. Previous studies have suggested that the more subjective measures, the TJC and VAS, may affect the ability of the 4C-DAS28 to detect ultrasound-determined synovitis and radiographic progression of joint damage and synovitis on MRI [11,14,15]. The 4C-DAS28 is a multivariable construct, which includes markers of inflammation, pain and patient assessment of disease activity. While the 4C-DAS28 was a significant achievement in the development of a DAS to homogenize patient assessment, TNFis are designed to target joint inflammation, and the 4C-DAS28 may not accurately measure joint inflammation alone. Refining the measure to more closely reflect ultrasound-determined synovitis may be a more appropriate measure for pharmacogenetic studies. This study aimed to investigate whether moving from the conventional 4C-DAS28 to the recently developed 2C-DAS28 can help approach the goal of improving prediction of treatment response using genetic biomarkers. The study demonstrated that when the absolute change in 2C-DAS28 was used to assess post-treatment disease activity at 6 months, more SNPs demonstrated association compared with the original 4C-DAS28. These findings are in keeping with a recent study that demonstrated that the reweighted 2C-DAS28 shows stronger association with ultrasound measured synovitis in three independent cohorts and radiographic progression of RA, when compared with the original 4C-DAS28 [11].

Studies have shown that there is a substantial degree of heritability in response to TNFi therapy [16] and that the heritability is higher for the more objective measures of the 4C-DAS28, the SJC and inflammatory blood markers [10]. A major limitation in previous pharmacogenetic TNFi treatment response studies may therefore have been the inclusion of the subjective (TJ28 and VAS) measures in the DAS28 calculation. In this study, more SNPs were associated with treatment response using the reweighted 2C-DAS28 than would have been expected by chance suggesting that it could be a preferred outcome measure in future clinical research in the assessment of disease response as it more accurately reflects ultrasound-determined synovitis.

Our study has some limitations that require discussion. First, due to the relatively modest sample size of this study, the power to detect associations may be limited. While previous studies regarding RA susceptibility loci have analyzed >100,000 samples, most TNFi response studies have used modest sample sizes (n <3000) [4,17]. In order to replicate previous associations, a relatively lenient statistical threshold of p ≤ 0.05 was chosen, increasing the risk of false-positive associations. Our aim however, was to validate association. Therefore, it is recommended that further research to validate the 2C-DAS28 associations in independent collections is undertaken. Classifying outcome according to response criteria may be more clinically relevant as opposed to change in DAS28 however, the majority of association studies identified in the literature review utilised change in 4C-DAS28 to define response (Supplementary Table 1). As response classification criteria for the 2C-DAS28 are yet to be defined, analysis was undertaken using multivariate linear regression, it is therefore not known whether patients would be reclassified as responders utilizing the 2C-DAS28 compared with the 4C-DAS28.

Second, pharmacogenomic studies are frequently collaborative and there is some overlap of samples analyzed here with previous studies [17–19] where we have collaborated. As part of Cui *et al.* 2010 [18] 81 patients and in Cui *et al.* 2013 [17] 140 patients were initially used to detect association with an additional 595 samples from BRAGGSS. The Plant *et al.* [19] study was undertaken in BRAGGSS patients. While this overlapmay inflate the Type 1 error, this would apply to both the 4C-DAS28 and the 2C-DAS28.

Third, lack of adherence to therapy inevitably translates to poorer clinical outcomes which can also have an impact on the analysis of treatment response as patients genetically predicted to respond are unlikely to do so if they do not adhere to their treatment [20]; this has not been adjusted for in this study or others before it.

At last, though RA can present relatively similarly in clinical presentation, it is possible that it is, in fact, a number of diseases with differing mechanisms. To illustrate the latter, studies have demonstrated that there are significant genetic and clinical differences between anticitrullinated protein antibody positive and negative RA patients [21]. This study has not differentiated between subsets of RA which would have reduced the power to identify genetic association.

## Conclusion

This study has replicated five SNPs associated with TNFi treatment response when using the validated 2C-DAS28 (as opposed to one with the conventional 4C-DAS28). With the importance of early treat to target approaches, the major aim of future research is to identify a molecular biomarker that will enable prediction of TNFi treatment response in RA. Taking a precision medicine approach, patients would ideally be stratified into treatment response groups and treated accordingly, thereby avoiding the current trial and error process of treatment optimization. Future research should consider using the 2C-DAS28 as a disease outcome measure in pharmacogenetic RA studies which more accurately reflects joint inflammation and may identify hitherto undiscovered associations.

**Summary points**

- TNF inhibitors (TNFi) are treatment options for patients with rheumatoid arthritis (RA).
- Approximately 40% of RA patients experience TNFi failure.
- There is a substantial degree of heritability in response to TNFi treatment.
- Replication of SNPs associated with TNFi response is partial with many SNPs failing to replicate.
- Response to TNFi is measured using the 4C-DAS28 which includes more subjective measures that can be affected by other illnesses such as depression.
- The 2C-DAS28 has been developed to more closely represent joint inflammation.
- Utilizing the 2C-DAS28 more SNPs are significantly replicated with association compared with the 4C-DAS28.
- Researchers should consider using the 2C-DAS28 as a disease outcome measure in pharmacogenetic RA studies.
