# Supplementary material for: Pharmacogenetics of TNF inhibitor response in rheumatoid arthritis utilizing the two-component disease activity score
Source: Pharmacogenomics. 2020 Oct 30;21(16):1151–6. doi: 10.2217/pgs-2020-0043 (PMC7649675; doi:10.2217/pgs-2020-0043)
Supplement: Supplementary file 1 [file pgs-21-1151-s1.docx]

Members of the Biologics in Rheumatoid Arthritis Genetics and Genomics Study Syndicate (BRAGGSS)

Steering Committee members: Professor Anne Barton, Professor John Isaacs, Professor Ann Morgan, Professor Gerry Wilson

Barts Health NHS Trust (Prof C Pitzalis)

Basingstoke & North Hampshire NHS Foundation Trust (Dr E Williams, Dr R K Moitra, Dr D J Shawe)

Burton Hospitals NHS Foundation Trust (Dr M Nisar)

Central Manchester University Hospitals NHS Foundation Trust - Manchester Royal Infirmary (Prof I Bruce, Prof A Barton, Dr R Gorodkin, Dr P Ho, Dr K Hyrich) - Trafford General (Dr F McKenna)

Chesterfield Royal Hospital NHS Foundation Trust (Dr K Fairburn)

Countess of Chester Hospital NHS Foundation Trust (Dr J Nixon, Dr T Barnes, Dr M Hui)

City Hospitals Sunderland NHS Foundation Trust (Dr D Coady, Dr D Wright, Dr C Morley, Dr G Raftery, Dr C Bracewell)

County Durham and Darlington NHS Foundation Trust (Dr R Reece, Dr. D. Armstrong, Dr. A J Chuck, Dr. S Hailwood, Dr N Kumar, Dr D Ashok,)

Derby Hospitals NHS Foundation Trust (Dr. S C O'Reilly, Dr T Ding, Dr. L J Badcock, Dr. C M Deighton , Dr N Raj, Dr. M R Regan, Dr. G D Summers, Dr. R A Williams)

Gateshead Health NHS Foundation Trust (Dr. C A Kelly, Dr. J Hamilton, Dr. C R Heycock, Dr V Saravanan)

Harrogate & District NHS Foundation Trust (Dr M Green, Dr A Gough, Dr C Lawson)

Kettering General Hospital NHS Foundation Trust (Dr A Kuttikat, Dr D Parthajit, Dr E Borbas, Dr T Wazir)

Leeds Teaching Hospitals NHS Trust (Prof. P Emery, Dr. S. Bingham, Prof. A. Morgan, Prof H A Bird, Prof P G Conaghan, Dr C T Pease, Dr R J Wakefield, Prof. M Buch, Dr S Dass)

Northumbria Healthcare NHS Foundation Trust (Dr F N Birrell, Dr P R Crook)

Northern Lincolnshire and Goole Hospitals NHS Foundation Trust (Dr B Szebenyi, Dr D Bates, Dr D James, Dr T Gillott, Dr A Alvi, C Grey, J Browning

Nottingham University Hospitals NHS Trust (Dr. J F McHale, Dr. I C Gaywood, Dr. A C Jones, Dr. P Lanyon, Dr. I Pande, Prof. M Doherty, Dr. A Gupta, Dr. P A Courtney, Dr A Srikanth, Dr A Abhishek)

Pennine Acute Hospitals NHS Trust – - North Manchester General Hospital (Dr L Das, Dr. M Pattrick, Dr. H N Snowden, Dr A P Bowden, Dr E E Smith, Dr P Klimiuk, Dr D J Speden ) - Fairfield Hospital (Dr L Das)

Portsmouth Hospitals NHS Trust (Dr. J M Ledingham , Dr. R G Hull, Dr. F McCrae, Dr. A Cooper, Dr S A Young Min, Dr Wong, Dr Shaban)

Royal Cornwall Hospitals NHS Trust (Prof A D Woolf, Dr M Davis, Dr D Hutchinson, Dr A Endean)

Royal Liverpool and Broadgreen University Hospitals NHS Trust (Dr D Mewar, Dr E J Tunn, Dr K Nelson, Dr T D Kennedy, Dr C Dubois)

Royal National Hospital for Rheumatic Diseases NHS Foundation Trust (Dr J Pauling, Dr E Korendowych, Dr T Jenkinson, Dr R Sengupta, Dr A Bhalla, Prof N McHugh, Dr W Tillett, Dr T Ahmed)

Salford Royal NHS Foundation Trust (Dr. H Chinoy, Prof T O’Neil, Prof A Herrick, Prof A Jones, Dr R Cooper, Dr W Dixon, Dr B Harrison)

Sheffield Teaching Hospitals NHS Foundation Trust (Dr. M Akil, Dr. S Till, Dr L Dunkley, Dr R Tattersall, Dr R Kilding, Dr T Tait, Dr J Maxwell, Dr K-P Kuet)

South Tees Hospitals NHS Foundation Trust (Dr. M J Plant, Dr. F Clarke, Dr. J N Fordham, Dr S Tuck, Dr S K Pathare, Dr A Paul)

South Warwickshire General Hospital NHS Trust (Dr. C P Marguerie, Dr S P Rigby, Dr N Dunn)

Staffordshire & Stoke-on-Trent Partnership NHS Trust (Dr S Hider, Dr A Menon, Dr C Dowson, Dr S Dutta, Dr S Kamath, Dr J Packham, Dr S Price, Dr E Roddy, Dr Z Paskins, Professor A. Hassell)

Stockport NHS Foundation Trust (Dr A Ismail, Dr C Filer)

St Helens and Knowsley Hospitals NHS Trust (Dr. R Abernethy, Dr A R Clewes, Dr. J K Dawson)

The Dudley Group of Hospitals NHS Foundation Trust (Prof G Kitas, Dr N Erb, Dr R Klocke, Dr A J Whallett, Dr K Douglas, Dr A Pace, Dr R Sandhu, Dr H John)

The Ipswich Hospital NHS Trust (Dr S Lane)

The Newcastle upon Tyne Hospitals NHS Foundation Trust (Prof. J D Isaacs, Prof. H Foster, Dr. B Griffiths, Dr. I Griffiths, Dr L Kay, Dr W-F Ng, Dr. P N Platt, Dr. D J Walker, Dr P Peterson, Dr A Lorenzi, Dr M Friswell, Dr B Thompson, Dr M Lee, Dr A Pratt)

The Royal Wolverhampton Hospitals NHS Trust - Cannock Chase Hospital (Dr. D Mulherin, Dr. S V Chalam, , Dr. T Price, Dr. T Sheeran, Dr S Venkatachalam, Dr S Baskar) - New Cross Hospital (Dr Sabrina Raizada)

University Hospital Birmingham NHS Foundation Trust (Dr A Filer, Dr. Bowman, Dr. P Jobanputra Dr. E C Rankin)

University Hospitals of Coventry and Warwickshire NHS Trust (Dr S Dubey, Dr. K Chaudhuri, Dr A Price-Forbes, Dr J Ravindran)

University Hospitals of Leicester NHS Trust (Dr A Moorthy, Dr P Sheldon, Dr W Hassan, Dr J Francis, Dr A Kinder, Dr R Neame)

University Hospitals of Morcambe Bay NHS Trust - Royal Lancaster Hospital (Dr. M Bukhari, Dr L Ottewell, Dr Palkonyai) - Furness General Hospital (Dr M Bukhari)

West Suffolk Hospitals NHS Trust (Dr D T O’Reilly, Dr V Rajagopal,)

Wrightington, Wigan and Leigh Hospitals NHS Foundation Trust (Dr E Gladston Chelliah)

York Teaching Hospitals NHS Foundation Trust - York District Hospital (Dr M Green, Dr M Quinn, Dr A Isdale, Dr A Brown, Dr B Saleem) - Scarborough Hospital (Dr Z Al-Saffar, Dr G Koduri)
